# Maternal Salivary Glutamate Concentrations in Cesarean Delivery: A Prospective Comparison Between Spinal and General Anesthesia

**DOI:** 10.3390/life16050748

**Published:** 2026-05-01

**Authors:** Diab Bani Hani, Omar F. Altal, Atef F. Hulliel, Tala Ahmed, Anas Alrusan, Mohammad Al Hazaymeh, Fatima Albusta, Taqwa Haweeleh, Ala”a Alhowary

**Affiliations:** 1 Department of Anesthesia, Faculty of Medicine, Jordan University of Science and Technology, Irbid 21110, Jordan; amalrusan@just.edu.jo (A.A.); mkalhazaymeh@just.edu.jo (M.A.H.); fatimaalbusta@hotmail.com (F.A.); t7aweleh@gmail.com (T.H.); alaacom78@yahoo.com (A.A.); 2 Department of Obstetrics and Gynecology, Faculty of Medicine, Jordan University of Science and Technology, Irbid 21110, Jordan; altal_omar@yahoo.com; 3 Faculty of Medicine, Jordan University of Science and Technology, Irbid 21110, Jordan; afhulliel181@med.just.edu.jo (A.F.H.); talasuhail293@gmail.com (T.A.)

**Keywords:** spinal anesthesia, general anesthesia, cesarean section, ELISA

## Abstract

Background: Cesarean delivery is commonly performed under either spinal or general anesthesia. While both techniques are widely used, their differential effects on maternal neurochemical profiles, particularly excitatory neurotransmitters such as glutamate, remain incompletely understood. Glutamate plays a central role in stress response, nociception, and neuroendocrine regulation. This study aimed to compare maternal salivary glutamate concentrations between spinal and general anesthesia during cesarean delivery. Method: In this prospective study, 66 women undergoing cesarean delivery were included: 32 received spinal anesthesia and 34 received general anesthesia. Unstimulated saliva samples were collected perioperatively and analyzed using a colorimetric enzymatic assay. Clinical and demographic variables were extracted from electronic medical records. Results: Baseline maternal and neonatal characteristics were comparable between groups (all *p* > 0.05). Glutamate concentrations were not normally distributed. Median salivary glutamate levels were significantly higher in the general anesthesia group compared with the spinal anesthesia group (8.05 nmol/µL [IQR 4.81–11.69] vs. 5.19 nmol/µL [IQR 3.05–8.39]; *p* = 0.041) After adjustment for maternal and perinatal covariates, anesthesia type was not independently associated with glutamate levels (*p* = 0.21). Conclusion: General anesthesia was associated with higher unadjusted maternal salivary glutamate levels compared with spinal anesthesia during cesarean delivery; however, this association did not persist after multivariable adjustment. These findings suggest that anesthesia technique alone may not independently influence perioperative glutamatergic responses. Further research is warranted to clarify the clinical significance of these neurochemical changes for maternal and neonatal outcomes.

## 1. Introduction

Cesarean delivery is one of the most frequently performed surgical procedures worldwide, with the choice of anesthetic technique, typically spinal or general anesthesia, playing a critical role in both maternal and neonatal outcomes [1]. While spinal anesthesia is often preferred for elective cases due to its rapid onset and reduced risk of airway complications, general anesthesia remains essential in emergent situations or when neuraxial techniques are contraindicated [2]. Despite the widespread use of these methods, their differential impact on maternal metabolic and neurochemical profiles, particularly regarding excitatory neurotransmitters like glutamate, remains an area of interest.

Glutamate is the primary excitatory neurotransmitter in the central nervous system and plays a role in various physiological and pathological processes [3]. During pregnancy, maternal glutamate concentrations are regulated, as excessive levels have been implicated several adverse effects [4]. Interestingly, research has demonstrated that fetal blood glutamate concentrations are significantly higher than maternal levels, suggesting a complex placental transport mechanism that may serve a neuroprotective function for the developing fetus [5]. The ability to modulate maternal glutamate levels through anesthetic intervention could therefore have significant implications for perioperative maternal and fetal well-being. For instance, disruption of glutamate homeostasis, whether through excess or deficiency, has been associated with excitotoxic neuronal injury, mediated by excessive calcium influx, mitochondrial dysfunction, oxidative stress, and ultimately cell death [6].

Understanding the neurobiological consequences of altered glutamate levels during the peripartum period is of particular importance. Glutamate-mediated excitotoxicity, resulting from excessive extracellular glutamate accumulation, is a recognized mechanism of neuronal injury implicated in perinatal brain damage, including periventricular white matter injury and hypoxic–ischemic encephalopathy [7].

In the developing fetal brain, immature oligodendrocytes and selected neuronal populations are especially vulnerable to glutamate-induced injury due to the transient developmental upregulation of glutamate transporters and the enhanced activity of calcium-permeable glutamate receptors during mid-to-late gestation [8]. In the peripartum context, glutamate concentrations in the medial prefrontal cortex are influenced by ovarian hormone fluctuations, and higher levels have been observed in women with postpartum depression compared with healthy postpartum controls [9].

The choice of anesthetic agent has been shown to influence systemic glutamate concentrations. For instance, volatile anesthetics used in general anesthesia, such as isoflurane, have been associated with significant and reversible increases in plasma glutamate levels in surgical patients [10]. This effect may be mediated by the modulation of glutamate transporters, which are sensitive to the pharmacological properties of various anesthetic drugs [11,12]. In contrast, spinal anesthesia, which primarily involves the intrathecal administration of local anesthetics, may exert different effects on the systemic and cerebrospinal fluid concentrations of excitatory amino acids.

Given the potential neurobiological consequences of altered glutamate levels during the peripartum period, understanding how different anesthetic techniques affect maternal glutamate concentrations is important. This prospective study aims to compare the effects of spinal and general anesthesia on maternal glutamate concentrations during Cesarean delivery, providing further insight into the neurochemical changes associated with these common anesthetic practices.

## 2. Methods and Materials

### 2.1. Patients and Data Collection

This prospective cohort study was conducted at King Abdullah University Hospital (KAUH), Irbid, Jordan, between January 2024 and January 2025, and included women undergoing CS. Participants were categorized according to the type of anesthesia administered: spinal anesthesia and general anesthesia. The primary comparison was between spinal and general anesthesia groups. Clinical and demographic data, including maternal age, body mass index (BMI), gestational age at delivery, neonatal birth weight, presence of gestational diabetes mellitus (GDM), preeclampsia, and neonatal intensive-care-unit (NICU) admission, were extracted from medical records to assess baseline group comparability and strengthen the internal validity of the primary glutamate analysis. In addition, BMI and neonatal birth weight were included as covariates in the regression analysis due to their potential influence on metabolic and inflammatory pathways relevant to glutamate metabolism and to evaluate potential confounders. GDM and preeclampsia were included as maternal comorbidities of interest, as both are associated with metabolic and vascular disturbances that may influence glutamate homeostasis. Participants with incomplete clinical data or missing saliva samples were excluded from analysis.

Women eligible for inclusion in this study were those who delivered through CS and who had received either spinal or general anesthesia. They were eligible for enrollment if they were at least 18 years of age, American Society of Anesthesiologists physical status 2, and with a single or twin gestation at 37–42 weeks of gestation. The exclusion criteria comprised a history of nongestational diabetes mellitus and chronic advanced renal disease. Moreover, women with a chronic pain syndrome, chronic use of pain medication, and antidepressants were excluded. Furthermore, all cases of spinal anesthesia converted to general anesthesia were excluded. Any women with absolute contraindications for either the spinal or general anesthesia were also excluded.

### 2.2. Outcomes, Patients’ Groups, and Allocation

The participants were allocated based on their choices and preferences. Pregnant women were categorized into two groups: women who received spinal anesthesia and women who received general anesthesia. The primary outcome was to measure the difference in the mean salivary glutamate levels at the induction of anesthesia, at the CS incision, and at skin closure. Secondary outcomes were to investigate the factors (maternal and medical) affecting these salivary glutamate levels in the mothers.

### 2.3. Anesthetic Setting

Consultant anesthesiologists and senior residents carried out and supervised the conduction of anesthesia. In the theater, two intravenous cannulas were inserted, and continuous monitoring of blood pressure, oxygen saturation, respiratory rate, and electrocardiogram were conducted.

#### 2.3.1. Spinal Anesthesia

At the level of L3–L4 or L4–L5 of the vertebral column, the SA was conducted under aseptic technique using 25- or 27-gauge spinal needles with 2.3 mL of 0.5% heavy bupivacaine and 0.4 mL of 0.005% fentanyl. Adequate hydration with crystalloid solution was performed before the procedure along with 100% O_2_ through a nasal cannula.

#### 2.3.2. General Anesthesia

Rapid-sequence induction was done with the insertion of an endotracheal tube sized 6.5–7.5 mm, and the induction was performed using propofol and rocuronium. Anesthesia was maintained using isoflurane in 50% oxygen and 50% air. After delivery of the baby and cutting the umbilical cord, fentanyl was given, inhaled anesthetic agents were discontinued and anesthesia was maintained with a propofol infusion. At the end of surgery, neostigmine and atropine were given intravenously.

### 2.4. Obstetrical Settings and Samples Collection

The CS operations were performed by a single consultant obstetrician. Foley’s catheter was inserted. Lower-segment transverse uterine incision was carried out. After delivery of the baby, all women received intravenous 10 IU oxytocin bolus and 20 IU oxytocin infusion over 1 h. Both groups were given 2–3 L of crystalloids fluid. The newborn was examined immediately by pediatricians for Apgar score and for the baseline perinatal examination.

Unstimulated saliva samples were collected from each participant during active labor or cesarean delivery using sterile collection tubes. Samples were immediately placed on ice, transported to the Aurum Biotech Diagnostic Laboratory (Amman, Jordan), and centrifuged at 3000× *g* for 15 min to remove debris. The clarified supernatant was aliquoted and stored at −80 °C until analysis.

### 2.5. Glutamate Quantification

Glutamate levels were quantified using the Abcam Glutamate Assay Kit (ab83389, Cambridge, UK) in accordance with the manufacturer’s instructions. This assay provides sensitive detection of free glutamate in biological samples and does not measure glutamate incorporated into peptides or proteins. The method is based on an enzymatic colorimetric reaction, in which glutamate is recognized as a specific substrate by the enzyme mix, leading to proportional color development.

Briefly, standards and appropriately diluted samples were added to 96-well plates, followed by the addition of the reaction mix containing the glutamate enzyme and developer. Plates were incubated for 30 min at 37 °C, after which absorbance was measured at 450 nm using a microplate reader (Bio-Tek Instruments, Winooski, VT, USA). Glutamate concentrations were calculated from a standard curve generated with the provided glutamate standard.

Samples were diluted as necessary to fall within the linear range of the assay. All samples were analyzed in duplicate, and mean values were used for statistical analysis. Blanks and quality control samples were included on each plate to ensure assay reliability and reproducibility.

### 2.6. Statistical Analysis

Data were entered into a spreadsheet and analyzed using IBM SPSS Statistics for Windows, Version 26.0. Nominal variables were expressed as frequency and percentage, while continuous variables were reported as mean ± standard error of the mean (SEM). Comparisons between groups were performed using the Chi-square test for categorical variables and Student’s *t*-test for continuous variables. A *p*-value ≤ 0.05 was considered statistically significant.

## 3. Results

### 3.1. Patient Demographics

A total of 66 women were included in the final analysis, with 32 (48.5%) receiving spinal anesthesia and 34 (51.5%) receiving general anesthesia. Baseline maternal and perinatal characteristics were similar between the two groups. No statistically significant differences were observed in maternal age, body mass index (BMI), gestational age at delivery, or neonatal birth weight (all *p* > 0.05). Similarly, the rates of gestational diabetes mellitus (GDM), placenta previa spectrum, antenatal steroid use, and neonatal intensive care unit (NICU) admission were comparable between groups. Notably, no cases of preeclampsia were identified in either group. These results indicate that the spinal and general anesthesia cohorts were well matched in terms of clinical and demographic factors, supporting a valid comparison of glutamate concentrations between the two anesthesia modalities (Table 1).

### 3.2. Comparison of Glutamate Levels

**Figure 1:**
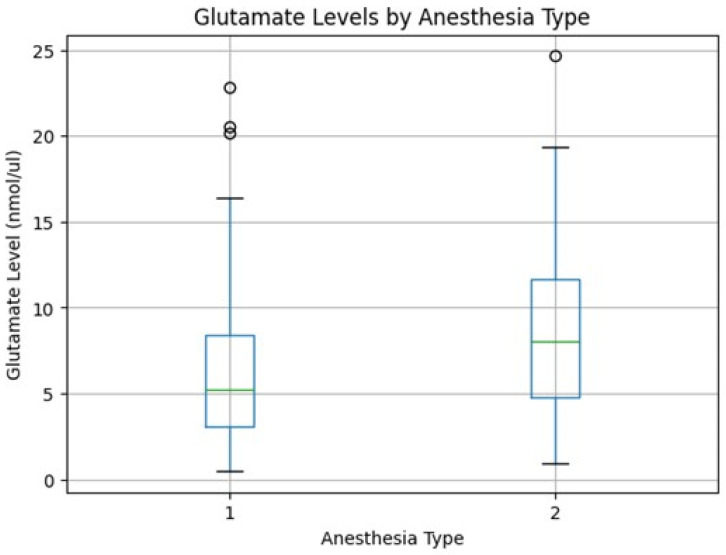
Boxplot of Glutamate levels by anesthesia type.

Normality testing using the Shapiro–Wilk test demonstrated that glutamate concentrations were not normally distributed in either anesthesia group (spinal anesthesia: *p* < 0.001; general anesthesia: *p* = 0.032). Accordingly, non-parametric analysis was applied. Median glutamate levels were significantly higher in the general anesthesia group compared with the spinal anesthesia group (8.05 nmol/µL [IQR 4.81–11.69] vs. 5.19 nmol/µL [IQR 3.05–8.39], respectively). This difference reached statistical significance (Mann–Whitney U = 384.0, *p* = 0.041). The distribution of glutamate values was right-skewed in both groups, supporting the use of non-parametric testing. A boxplot illustrating the distribution of glutamate levels by anesthesia type is shown in Figure 1 (Table 2).

### 3.3. Secondary Analyses

To assess the influence of anesthesia type and selected maternal and perinatal variables on glutamate concentrations, a multiple linear regression model was constructed using log-transformed glutamate levels as the dependent variable. Independent variables included anesthesia type, maternal age, BMI, gestational age at delivery, neonatal birth weight, and GDM status. After adjustment for all covariates, anesthesia type was not independently associated with log-transformed glutamate levels (*p* = 0.21). None of the other examined maternal or perinatal variables demonstrated a statistically significant association with glutamate concentrations (all *p* > 0.48). The overall model explained a limited proportion of the variability in glutamate levels (R^2^ = 0.11; adjusted R^2^ = −0.06), suggesting that factors beyond those included in the model may contribute to inter-individual differences in glutamate concentrations (Table 3).

### 3.4. Factors Affecting Glutamate Levels

Among the evaluated maternal, obstetric, and anesthetic factors, no variable demonstrated an independent statistically significant association with glutamate concentrations in multivariable analysis. Although glutamate levels differed between anesthesia groups in univariate analysis, this association did not persist after adjustment for potential confounders, indicating that anesthesia type alone was insufficient to explain variability in glutamate levels.

### 3.5. Distribution of Glutamate Levels

**Figure 2:**
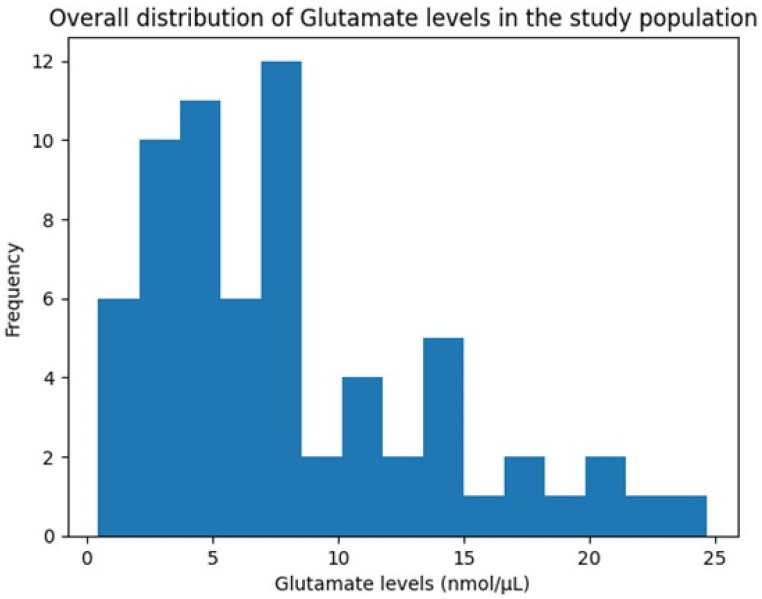
Histogram showing the overall distribution of Glutamate levels (nmol/µL).

The overall distribution of glutamate concentrations across the study population demonstrated considerable inter-individual variability, with values ranging from 0.46 to 24.68 nmol/µL and a pronounced right-skewed pattern (Figure 2). This distribution suggests heterogeneous glutamatergic responses during childbirth, potentially reflecting differences in physiological stress responses or metabolic regulation.

## 4. Discussion

Glutamate, a non-essential amino acid, serves as the principal excitatory neurotransmitter within the central nervous system and is the most abundant neurotransmitter in the brain [13,14].

Beyond its role in neurotransmission, glutamate is critical for various physiological processes, including metabolic pathways and cellular energy production [15]. Its wide presence and diverse functions underscore its importance in maintaining normal brain function and overall physiological homeostasis. The intricate balance of glutamate levels is tightly regulated, as both insufficient and excessive concentrations can lead to significant neurological dysfunction and excitotoxicity [15,16].

As a neurotransmitter, glutamate mediates fast excitatory synaptic transmission by acting on a variety of receptors, including ionotropic N-methyl-D-aspartate (NMDA), alpha-amino-3-hydroxy-5-methyl-4-isoxazolepropionic acid (AMPA), and kainate receptors, as well as metabotropic glutamate receptors (mGluRs) [15]. This diverse receptor profile allows glutamate to exert a wide range of effects on neuronal excitability and synaptic plasticity, which are fundamental for cognitive functions such as learning and memory.

Surgical procedures induce a profound systemic stress response, characterized by the activation of the hypothalamic–pituitary–adrenal (HPA) axis and the sympathetic nervous system [17,18]. This neuroendocrine response leads to a cascade of physiological changes, including alterations in circulating neurotransmitter levels. Notably, surgical stress and trauma have been shown to increase circulating glutamate concentrations [10].

This study investigated the differential impact of spinal versus general anesthesia on maternal salivary glutamate concentrations during Cesarean delivery. Our primary finding reveals that patients undergoing general anesthesia exhibited significantly higher median salivary glutamate levels compared to those receiving spinal anesthesia.

In our study, we observed significantly higher median salivary glutamate levels in patients undergoing general anesthesia (8.05 nmol/µL) compared to those receiving spinal anesthesia (5.19 nmol/µL), with this difference reaching statistical significance (*p* = 0.041). This finding aligns with existing literature suggesting that general anesthesia can elevate circulating glutamate levels. For instance, Stover and Kempski (2005) reported increased plasma glutamate in neurosurgical patients under isoflurane anesthesia, supporting the notion that general anesthetics can impact glutamate homeostasis [10]. The observed elevation in the general anesthesia group in our study may reflect a more pronounced systemic stress response or direct effects of general anesthetic agents on glutamate metabolism and transport mechanisms, consistent with studies showing volatile anesthetics can modulate EAAT activity [11] which is responsible for the majority of glutamate uptake in the brain and peripheral tissues. Conversely, the comparatively lower glutamate levels in the spinal anesthesia group in our univariate analysis could be attributed to the regional technique’s ability to attenuate the systemic neuroendocrine stress response, as highlighted by reviews on regional anesthesia’s influence on stress markers [19].

The relationship between glutamate levels and pain processing is a critical aspect of perioperative neurobiology. Glutamate is the primary neurotransmitter involved in the transmission of nociceptive information from the periphery to the central nervous system [20]. Upon tissue injury, such as that occurring during a Cesarean section in our paper, primary afferent fibers release glutamate into the dorsal horn of the spinal cord [21]. This release activates ionotropic receptors, particularly NMDA and AMPA receptors, which are essential for the development of central sensitization and hyperalgesia [22]. Research has shown that elevated levels of glutamate in the spinal cord and peripheral tissues are directly correlated with the intensity of acute pain [23]. In the context of labor and delivery, studies have identified changes in excitatory amino acids in the cerebrospinal fluid, suggesting that the glutamatergic system is actively involved in labor pain modulation [23]. Furthermore, salivary glutamate has emerged as a potential non-invasive biomarker for pain and stress, with elevated levels observed in conditions such as chronic migraine [24]. The significantly higher glutamate levels observed in our general anesthesia group may therefore reflect a higher degree of physiological “pain stress” or a less effective suppression of the glutamatergic signaling cascade compared to spinal anesthesia.

Recent evidence suggests that salivary glutamate also reflects neuroendocrine stress, with increased levels reported in individuals experiencing anxiety, depression, and chronic stress [25]. During pregnancy, maternal glutamate concentrations are tightly regulated to ensure fetal neuroprotection, as the placenta actively manages the transport of glutamate between the maternal and fetal circulations [5]. Our findings suggest that the choice of anesthesia during Cesarean delivery can influence this delicate maternal neurochemical profile, potentially impacting the perioperative environment for both mother and neonate.

In conclusion, our study demonstrates that general anesthesia is associated with significantly higher salivary glutamate levels compared to spinal anesthesia during Cesarean delivery. This difference likely reflects the varying degrees of stress response modulation and the direct pharmacological effects of anesthetic agents on the glutamatergic system. While spinal anesthesia appears to maintain lower systemic glutamate levels, potentially indicating a more effective blunting of the surgical stress response, further research is needed to elucidate the long-term clinical implications of these neurochemical changes on maternal recovery and neonatal outcomes.

This study has several limitations that should be acknowledged. First, the sample size was relatively small (n = 66), which may have limited statistical power. Second, anesthesia type was based on clinical indication and patient preference rather than randomization, introducing potential selection bias. Lastly, as an observational study, we accounted for several potential confounders; however, the presence of residual confounding cannot be ruled out.

Despite these limitations, the study has several strengths. To our knowledge, it is among the first to compare salivary glutamate concentrations between spinal and general anesthesia during cesarean delivery. The prospective design, standardized anesthetic and surgical protocols performed by the same consultant teams, and the use of a validated colorimetric enzymatic assay support the reliability of our findings. Additionally, the multivariable analysis allowed assessment of the independent contribution of anesthesia type beyond unadjusted comparisons.

## 5. Conclusions

This prospective study demonstrated that women undergoing cesarean delivery under general anesthesia exhibited significantly higher median salivary glutamate concentrations compared with those receiving spinal anesthesia in unadjusted analysis. These findings are consistent with the hypothesis that general anesthesia may be associated with a more systemic stress response or direct modulation of glutamatergic pathways.

However, after adjustment for relevant maternal and perinatal variables, anesthesia type was not independently associated with glutamate levels. This suggests that the observed differences may be partially influenced by confounding physiological factors rather than anesthesia modality alone.

Taken together, these results indicate that anesthetic technique may contribute to alterations in maternal glutamatergic activity during cesarean delivery, but its independent effect appears limited. Larger studies incorporating longitudinal measurements and neonatal outcomes are needed to better understand the clinical relevance of perioperative glutamate fluctuations and their potential implications for maternal recovery and fetal neuroprotection.

## Figures and Tables

**Table 1:** Baseline Maternal and Clinical Characteristics by Anesthesia Type.

Variable	Spinal	General	*p* Value
Maternal age (years)	32.30 ± 5.30	33.99 ± 4.52	0.169
BMI (kg/m^2^)	28.44 ± 2.84	29.11 ± 3.73	0.520
Gestational age at delivery (weeks)	37.04 ± 1.98	36.63 ± 1.72	0.373
Fetal birth weight (kg)	2.71 ± 0.53	2.84 ± 0.59	0.357
Gestational diabetes mellitus	2 (6.2%)	2 (5.9%)	0.999
Placenta previa spectrum	0 (0.0%)	1 (2.9%)	0.999
Antenatal steroid use	14 (43.8%)	14 (41.2%)	0.999
NICU admission	4 (12.5%)	6 (17.6%)	0.811

**Table 2:** Distribution of Glutamate Levels by Anesthesia Type, using Median and IQR.

Group (Anesthesia)	N	Median	IQR (Q1–Q3)
Spinal	32	5.19	3.05–8.39
General	34	8.05	4.81–11.69

**Table 3:** Multiple linear regression model.

Predictor	β Coefficient	Standard Error	t Value	*p* Value	95% CI
Maternal age (years)	0.03	0.04	0.72	0.48	−0.05 to 0.10
BMI (kg/m^2^)	−0.04	0.06	−0.65	0.52	−0.16 to 0.09
Gestational age at delivery (weeks)	−0.01	0.15	−0.07	0.94	−0.32 to 0.29
Birth weight (kg)	−0.10	0.46	−0.23	0.82	−1.04 to 0.83
GDM (yes vs. no)	0.10	0.96	0.11	0.92	−1.86 to 2.06
Anesthesia type (general vs. spinal)	0.38	0.29	1.28	0.21	−0.22 to 0.97

## Data Availability

The original contributions presented in this study are included in the article. Further inquiries can be directed to the corresponding author(s).
